# New Insights Into the Phytochemical Composition and Biological Potential of Leaf Extracts From Algerian Autochthonous *Vitis vinifera* L.

**DOI:** 10.1002/fsn3.72214

**Published:** 2026-08-10

**Authors:** Radhia Belmanaa, Azzeddine Zeraib, Ziane Laiadi, Ahmed Simozrag, Khadra Afaf Bendrihem, Ayomide Victor Atoki, Mohammed Messaoudi

**Affiliations:** ^1^ Laboratory of Genetics, Biotechnology and Valorization of Bio‐Resources University of Biskra Biskra Algeria; ^2^ Biotechnology, Water, Environment and Health Laboratory, Faculty of Natural and Life Sciences University of Abbes Laghrour Khenchela Algeria; ^3^ Department of Biochemistry Kampala International University Ishaka Uganda; ^4^ Department of Chemistry, Higher Normal School of Kouba (ENS) Laboratory of Research on Bioactive Products and Biomass Valorization Algiers Algeria; ^5^ Department of Chemistry, Higher Normal School of Kouba (ENS) Laboratory N—Body and Structure of Matter Algiers Algeria

**Keywords:** antibacterial efficacy, antidiabetic, grape leaf extract, molecular docking, oxidative stress, phenolic compounds

## Abstract

*Vitis vinifera* leaves are abundant by‐products of the grape industry, often discarded as agricultural waste. This study explores the in vitro and in silico antioxidant, antidiabetic, and antibacterial properties of hydromethanolic crude extracts from grapevine leaves from five local grapevine varieties in Algeria (Chikki, Azorith‐fereinguel, Bezoul El‐khadem d'Algérie, Amesski El Hamma, and Hasram). Extracts were prepared via ultrasonic‐assisted extraction, with subsequent phytochemical profiling performed by liquid chromatography/mass spectrometry (LC/MS). The analysis revealed 17 identified phenolic compounds, primarily flavanols, and a few phenolic acids, including Isoquercitrin, Hesperidin, and Rutin, which were major compounds. Critically, the Azorith‐fereinguel variety exhibited superior antioxidant and antidiabetic activity (IC50: 3.93 μg/mL for DPPH; 43.61 μg/mL for ABTS; 75.12 μg/mL for RP; 22.46 μg/mL for α‐amylase; and 25.30 μg/mL for α‐glucosidase), outperforming many standard natural extracts and approaching the efficacy of synthetic controls. Furthermore, three varieties (Azorith‐fereinguel, Bezoul El‐khadem, Amesski El Hamma) showed potent antibacterial activity against four pathogens, with minimum inhibitory concentrations lower than standard antibiotics, indicating a potential solution for antibiotic resistance. In silico analyses revealed that hesperidin and rutin have high binding affinities to key enzyme active sites, providing a mechanistic basis for the observed bioactivity. These findings transform grapevine leaves from agricultural waste into a high‐value, multi‐functional natural product. This work provides a scientific foundation for developing grapevine leaf extracts into affordable, accessible nutraceuticals, functional food ingredients, or natural food preservatives for managing oxidative stress, diabetes, and bacterial infections, while simultaneously valorizing an abundant agro‐industrial by‐product within a circular food economy, particularly in resource‐limited settings where grape by‐products are abundant.

## Graphical abstract

Phenolic‐rich extracts from five indigenous Algerian *Vitis vinifera* leaf cultivars were characterized by LC–MS and evaluated for antioxidant, antidiabetic, antibacterial, and molecular docking activities. The Azorith‐fereinguel cultivar showed superior bioactivity, attributed mainly to isoquercitrin, hesperidin, and rutin. Docking analyses validated their interactions with therapeutic targets, highlighting grapevine leaves as sustainable agro‐industrial by‐products for developing nutraceuticals, functional foods, and natural food preservatives.

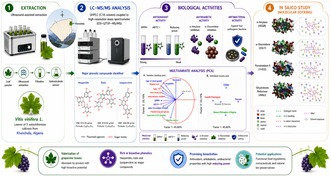

## 1 Introduction

*Vitis vinifera* L. (grapevine) is one of the most extensively cultivated fruit crops worldwide, with annual production exceeding 75 million metric tons (Fierascu et al. 2020). Beyond its primary role in wine and table grape industries, viticulture generates substantial phenolic‐rich by‐products, including leaves, stems, pomace, and seeds. Historically discarded or returned to soil as organic amendments, these residues are increasingly recognized as sustainable reservoirs of high‐value bioactive compounds aligned with circular bioeconomy and green chemistry principles (Castrica et al. 2019; Trigo et al. 2020; Osorio et al. 2021; Troilo et al. 2021). In Algeria, the largest country in North Africa, viticulture spans over 69,500 ha, yielding approximately 450,000 tons of grapes annually (OIV 2023). Despite its long‐standing viticultural heritage and remarkable agrobiodiversity, the systematic valorization of grapevine by‐products remains critically underdeveloped. Beyond their traditional medicinal uses, grapevine leaves are widely consumed as edible plant tissues in Mediterranean and Middle Eastern cuisines (e.g., as stuffed grape leaves/dolma), underscoring their direct relevance to human nutrition and food systems, and distinguishing them from purely medicinal plant materials (Acero et al. 2025).

Among grapevine residues, leaves have received comparatively less scientific attention than seeds, skins, or pomace, despite their historical use in traditional medicine for wound healing, anti‐inflammatory, and metabolic regulating purposes (Fernandes et al. 2013; Ramadan et al. 2017). Recent phytochemical investigations have confirmed that grapevine leaves are exceptionally rich in polyphenols, particularly flavonols (e.g., quercetin, rutin, and isoquercitrin) and phenolic acids, which exhibit potent antioxidant, antimicrobial, and enzyme‐inhibitory properties (Katalinić et al. 2013; Baroi et al. 2022; Acero et al. 2025). These compounds primarily act through radical scavenging, metal chelation, membrane stabilization, and targeted enzyme inhibition, positioning grapevine leaves as a promising natural alternative to synthetic additives and conventional pharmaceuticals.

However, the phytochemical composition and biological efficacy of grapevine leaves are highly dependent on genetic background, climatic stressors, soil composition, and phenological stage (Fernandes et al. 2013; Katalinić et al. 2013; Ghitti et al. 2022). Autochthonous cultivars, shaped by centuries of adaptation to semi‐arid Mediterranean and North African environments, often exhibit distinct secondary metabolite profiles compared to commercial clones (Laiadi et al. 2009; Riahi et al. 2010; Belmanaa et al. 2025). Despite this, the vast majority of existing literature focuses on European, American, or widely cultivated international varieties, leaving indigenous North African germplasm critically underexplored. This knowledge gap limits the development of region‐specific, standardized botanical extracts for nutraceutical and phytopharmaceutical applications.

Furthermore, while in vitro bioactivity screening provides initial evidence of plant extract efficacy, it rarely elucidates the underlying molecular mechanisms. The integration of computational approaches, particularly structure‐based molecular docking, has emerged as a powerful strategy to predict ligand–target interactions, validate experimental findings, and accelerate the discovery of plant‐derived therapeutics (Agu et al. 2023; Al‐Mijalli et al. 2025). Targeting key enzymes involved in oxidative stress (peroxiredoxin 5), carbohydrate metabolism (α‐amylase, α‐glucosidase), and bacterial folate biosynthesis (dihydrofolate reductase, DHFR) offers a multi‐dimensional framework to comprehensively assess the pharmacological potential of botanical extracts (Wróbel et al. 2020; Bayazeed et al. 2022; Pan and Kakeya 2025; Swargiary et al. 2025).

To address these gaps, this study presents the first integrated phytochemical and pharmacological profiling of five autochthonous *V. vinifera* leaf cultivars from the Khenchela region (Aurès Mountains) of northeastern Algeria. Specifically, we aim to (i) quantify total phenolic and flavonoid contents, (ii) identify and quantify key bioactive compounds using LC–MS, (iii) evaluate in vitro antioxidant, antidiabetic, and antibacterial activities, and (iv) validate observed bioactivities through in silico molecular docking against validated protein targets. By establishing cultivar‐specific phenolic fingerprints and correlating them with multi‐target bioactivities, this work provides a scientifically robust foundation for the sustainable valorization of Algerian grapevine leaves, supporting the development of standardized, evidence‐based botanical formulations for metabolic and infectious disease management.

## 2 Materials and Methods

### 2.1 Plant Material

Five indigenous grape varieties cultivated in the Khenchela region from Algeria (35°25′55″ N, 7°08′40″ E) were selected for this study. These varieties were characterized using a combined molecular and ampelographic approach (Belmanaa et al. 2025). Voucher specimens were deposited at the herbarium of the Laboratory of Genetics, Biotechnology and Valorization of Bio‐Resources, University of Biskra, Algeria, under accession codes GBVBR‐VV‐24‐01 (Chikki) to GBVBR‐VV‐24‐05 (Hasram) (Table 1). The cultivars exhibit berries with distinct pigmentation, encompassing red, black, green, and white colors. Leaves were collected in September during the maturation phase, following phenological stages defined by Baggiolini (1952). The varieties were designated using common regional names, which reflect traits such as color, size, morphology, or agronomic properties. For berry skin color and vine vigor, descriptors from the International Organization of Vine and Wine (OIV 2001) were utilized, with the modal value designated as the representative score for each parameter.

**TABLE 1:** Algerian autochthonous grape cultivars selected for this study.

Cultivars				Color		Growth		Common name meaning
Prime name	Common name	Voucher codes	VIVC ID	Grape color	OIV rating value	Vigorous growth	OIV rating value	
Chikki	Aghogalth	GBVBR‐VV‐24‐01	5186	Red	5	Very strong	9	Red, in amazigh languages
Azorith‐fereinguel	Abedaya	GBVBR‐VV‐24‐02	n.y.	White	1	Very strong	9	Early stage grape in Algerian Dialecte
Bezoul El‐khadem d'Algérie	Sbaa Laarous	GBVBR‐VV‐24‐03	24,150	Black	6	Medium	5	The fingers of the brid in Algerian dialecte
Amesski El Hamma	Anonymous	GBVBR‐VV‐24‐04	n.y.	Yellow	1	Medium	5	/
Hasram	Hasram	GBVBR‐VV‐24‐05	n.y.	Green	1	Medium	5	Used for the sour taste in Amazigh languages

Abbreviations: n.y., not yet registered in the VIVC database (pending submission for these newly characterized accessions); OIV, International Organization of Vine and Wine; VIVC, *Vitis* International Variety Catalogue.

### 2.2 Preparation of Extracts

Before extraction, the collected leaves were carefully dried in a well‐ventilated area away from direct light. The dried plant material was then mechanically ground into a fine powder (particle size ≤ 250 μm) to enhance the interaction between the constituents and the extraction solvent. In this study, ultrasonic‐assisted extraction was performed using a VCX 130 Ultrasonic Processor (Vibra‐Cell, USA). A hydromethanolic mixture (80:20 v/v) served as the extraction solvent. This ratio is widely documented to enhance the extraction of a broad polarity range of phenolic compounds compared to pure solvents (Bendrihem et al. 2024). Ultrasonic treatment was carried out for 25 min (130 W, 20 kHz) with a solid‐to‐liquid ratio of 1:20 (w/v) in cycles of 20 s “On” and 10 s “Off”. A cold bath maintained a constant temperature of 40°C (Insang et al. 2022). After extraction, the resulting extracts were centrifuged at 6000 rpm for 15 min, then filtered under vacuum using Whatman No. 1 filter paper. The filtrates were subsequently concentrated in triplicate using a rotary evaporator set at 40°C to ensure complete solvent removal. The obtained hydromethanolic extracts were stored in amber glass vials in the refrigerator at 4°C until the analysis.

### 2.3 Quantitative Determination of Chemical Constituents

#### 2.3.1 Total Phenolic Content

The total phenolic content (TPC) of grape leaf extracts was measured using a modified Folin–Ciocalteu colorimetric method described by Müller et al. (2010). In a 96‐well microplate, 20 μL of each extract (1 mg/mL) was mixed with 75 μL of a 7.5% sodium carbonate solution and 100 μL of Folin–Ciocalteu reagent (tenfold diluted in distilled water). After incubating the mixtures in the dark at room temperature for 2 h, absorbance was measured at 765 nm. TPC was calculated using a standard gallic acid curve, with concentrations ranging from 0 to 200 μg/mL, and the regression equation (*y* = 0.0034*x* + 0.1044, *R* ^2^ = 0.997).

#### 2.3.2 Total Flavonoids Content

The total flavonoid content (TFC) of grape leaf extracts was measured using the aluminum chloride colorimetric method, as described by Topçu et al. (2007), with minor modifications. In a 96‐well microplate, 50 μL of each extract (1 mg/mL) was mixed with 10 μL of potassium acetate (1 M), 10 μL of aluminum nitrate (10%), and 130 μL of methanol. The mixture was incubated at room temperature for 45 min. After incubation, the optical density was measured at 415 nm. A standard quercetin curve was prepared under the same conditions, with concentrations ranging from 0 to 50 μg/mL. TFC was calculated using the linear regression equation (*y* = 0.004*x*, *R* ^2^ = 0.997).

### 2.4 LC–MS Separation and Identification of Phenolic Compounds

For the LC/MS analysis, *V. vinifera* leaves extracts (VLEs) were prepared according to the method described by Griffith et al. (2019), with slight modifications. A 50 mg sample of the crude extract was dissolved in a mixture containing 1 mL of methanol and 1 mL of n‐hexane in a 2 mL Eppendorf tube. The mixture was vortexed using a Bioprep‐24 homogenizer for 2 min at 4°C, then centrifuged at 9000 rpm for 10 min at the same temperature using a Hettich Universal 320R centrifuge (Germany). The methanol phase was carefully separated and diluted with distilled water at a 1:9 ratio. The samples were then filtered through a Captiva Premium syringe filter consisting of a polypropylene shield and a nylon membrane with a 25 mm diameter and a 0.45 μm pore size, with an injection volume of 5.12 μL.

LC–MS/MS analysis was performed using an Agilent 1260 Infinity II LC System coupled to an Agilent 6460 Triple Quadrupole Mass Spectrometer. Chromatographic separation was achieved on a reversed‐phase Agilent Poroshell 120 EC‐C18 analytical column (100 mm × 3.0 mm, 2.7 μm). The flow rate was set at 0.40 mL/min and the column temperature was maintained at 40°C. The mobile phases consisted of Eluent A (water containing 5 mM ammonium formate) and Eluent B (acetonitrile containing 0.1% formic acid). Separation was performed using a gradient elution program as follows: 25% B (1–3 min), 50% B (4–12 min), 90% B (13–21 min), and 3% B (22–25 min), followed by re‐equilibration of the column before the next injection.

Mass spectrometric detection was carried out using electrospray ionization (ESI) operating in both positive and negative ionization modes. Data acquisition and processing were performed using Agilent MassHunter Software. Compound identification and quantification were achieved using a multiple reaction monitoring (MRM) approach, with collision energies optimized for each analyte to ensure efficient ion fragmentation and transmission. The capillary voltage was set at 4000 V, the gas temperature at 300°C, the nitrogen drying gas flow rate at 11 L/min, and the nebulizer pressure at 15 psi (Köktürk et al. 2022).

### 2.5 Antioxidant Assays

#### 2.5.1 DPPH Radical Scavenging Assay

The ability of VLEs to trap the DPPH radical was assessed using the method described by Blois (1958). A 40 μL mixture of the extracts or standards was combined with 160 μL of a one mM DPPH solution and allowed to stand for 30 min. Absorbance was then measured at 517 nm. The percentage inhibition was calculated using the following Equation (1), and the results were expressed as IC_50_ values:

(1) $$\text{Inhibition rate}\;\left(\%\right)=\left[\left(A_{c}-A_{s}\right)/A_{c}\right]\times 100$$

where *A*_c_ is the absorbance of control and *A*_s_ is the absorbance of samples.

#### 2.5.2 ABTS Radical Scavenging Assay

The radical scavenging ability of VLEs was evaluated using the method described by Re et al. (1999). ABTS^+^ radical cations were prepared by reacting 2 mM ABTS with 2.45 mM potassium persulfate in distilled water. The mixture was kept in the dark at room temperature for 16 h, then diluted to an absorbance of 0.700 ± 0.02 at 734 nm. For the assay, 40 μL of each crude extract (12.5–800 μg/mL) was mixed with 160 μL of the diluted ABTS solution in a 96‐well microplate. After a 10‐min incubation at room temperature, the absorbance was read at 734 nm. Positive controls included BHA, BHT, and α‐tocopherol. The inhibition percentage of the VLEs was estimated in terms of the ABTS radical cation decolorization according to the following Equation (1).

#### 2.5.3 Reducing Power Assay

For the measurement of the reductive ability, we investigated the Fe^3+^–Fe^2+^ transformations in the presence of VLEs following the method described by Oyaizu (1986). It was assessed by mixing 10 μL of each extract or standard (BHA, BHT and α‐Tocopherol) with 40 μL of phosphate buffer (0.2 M, pH 6.6) and 50 μL of potassium ferricyanide (K₃Fe(CN)₆, 1%) in a microplate. The mixture was incubated for 20 min at 50°C. Subsequently, 50 μL of trichloroacetic acid (C_2_HCl₃O_2_, 10%) was added to acidify the mixture, which was then mixed with 40 μL of distilled water and 10 μL of ferric chloride (FeCl₃, 0.1%). Absorbance was measured at 700 nm, and *A*_0.5_ values were recorded as results.

### 2.6 Enzyme Inhibition

α‐Amylase (from porcine pancreas, EC 3.2.1.1, Sigma‐Aldrich, St. Louis, MO, USA) and α‐glucosidase (from *Saccharomyces cerevisia*, EC 3.2.1.20, Sigma‐Aldrich) were used.

#### 2.6.1 α‐Amylase Inhibition Assay

The α‐amylase inhibitory activity of the VLEs was evaluated using the potassium iodine (KI) method (Yenigün et al. 2024). In a 96‐well plate, 82 μL of the extracts or acarbose at different concentrations was mixed with 10 μL of a 1 U α‐amylase solution (prepared in 20 mM potassium phosphate buffer, pH 6.9) in each well. The mixture was carefully combined and incubated at 37°C for 10 min. Subsequently, 8 μL of a 1% starch solution was added to each well and incubated at 37°C for a further 12 min.

Afterward, 50 μL of 10% hydrochloric acid (HCl) and 15 μL of iodine‐KI solution (composed of 2.5 mM iodine and 6.5 mM KI) were added. The mixture was then placed in boiling water for 10 min. The absorbance of each well was measured at 620 nm. The inhibition percentage was calculated and IC_50_ values (μg/mL) were calculated as follows:

(2) $$\text{Inhibition rate}\;\left(\%\right)=\left[\left(A_{0}–A_{1}\right)/A_{0}\right]\times 100$$

where *A*_0_ is the absorbance of control and *A*_1_ is the absorbance of samples.

#### 2.6.2 α‐Glucosidase Inhibition Assay

The α‐glucosidase inhibitory activity of the VLEs was evaluated using the 4‐p‐nitrophenyl α‐D‐glucopyranoside (p‐NPG) method, as described by Mayur et al. (2010). In a 96‐well plate, 10 μL of the extracts or acarbose at different concentrations was mixed with 25 μL of a 0.2 U α‐glucosidase solution prepared in 20 mM potassium phosphate buffer (pH 6.9). Subsequently, 25 μL of 0.5 mM p‐NPG solution and 20 μL of 20 mM potassium phosphate buffer (pH 6.9) were added to each well to ensure a homogeneous reaction mixture. The mixture was incubated at 37°C for 12 min, after which 100 μL of 0.2 M sodium carbonate (Na_2_CO₃) solution was added to each well and mixed thoroughly. The absorbance was measured at 410 nm, and IC_50_ values (μg/mL) were calculated to determine inhibitory activity.

### 2.7 Antibacterial Activity

The antibacterial activity was assessed using the minimum inhibitory concentration (MIC) method via broth microdilution (Zgoda and Porter 2001) against two Gram‐negative bacteria, *Pseudomonas aeruginosa* (ATCC 15442) and *Escherichia coli* (ATCC 25922), and two Gram‐positive bacteria, *Bacillus cereus* (CCM 99) and *Staphylococcus aureus* (ATCC 25213). All the tested bacterial strains were subcultured from the initial culture, stored at −70°C, maintained on Müller‐Hinton (MH) agar plates at 4°C, and cultivated at 37°C for 24 h.

In a sterile 96‐well plate, a series of dilutions of the extracts or antibiotics was prepared, starting at 1024 μg/mL and decreasing to 0.5 μg/mL. One milliliter of the bacterial solution, standardized to a 0.5 McFarland standard with a sterile saline solution (0.85% NaCl), was combined with 9 mL of MHB, and 5 μL of this mixture was added to each well. The plate was stored at +4°C for 2 h and incubated at 37°C for 18 h. After incubation, the agar plates were visually inspected for signs of growth or turbidity, and the results were recorded in μg/mL.

### 2.8 In Silico Investigation (Molecular Docking)

All molecular docking calculations and data visualization (including 2D interaction diagrams and 3D structural images) were performed using Molecular Operating Environment (MOE) 2015.10 software (Chemical Computing Group, Montreal, Canada).

#### 2.8.1 Ligands Preparation

The three‐dimensional structures of all the compounds characterized from the grapevine leaves of the studied varieties were obtained from the PubChem platform and saved as SDF files. In addition to the standard drugs (acarbose, BHA, BHT, and α‐tocopherol), these structures were transferred to the MOE system to create the database. Polar and nonpolar hydrogens were added, energy minimization was performed using the MMFF94x force field, partial charges were calculated, and the data was saved as an mdb file extension.

#### 2.8.2 Target Proteins Preparation

Human intestinal α‐glucosidase and pancreatic α‐amylase were selected as protein targets for antidiabetic assays, dihydrofolate reductase (DHFR) for antibacterial activity, and human peroxiredoxin 5 for the antioxidant test. The three‐dimensional crystal structures of the target enzymes were retrieved from the Protein Data Bank (PDB): 2QLY (α‐glucosidase), 4GQR (α‐amylase), 4M6J (DHFR), and 1HD2 (peroxiredoxin 5), deposited by Sim et al. (2008), Williams et al. (2012), Bhabha et al. (2013), and Declercq et al. (2001), respectively. The enzymes were prepared using the MOE software by removing heteroatoms and water molecules, adding hydrogen atoms, assigning correct bond orders, and optimizing the protonation states of the amino acids. Subsequently, the structures were subjected to energy minimization using the Amber99 force field.

#### 2.8.3 Validation of the Docking Method

To validate the accuracy of the docking protocol, the co‐crystallized ligands were re‐docked into the target enzyme's binding site. Docking results are deemed reliable when the root‐mean‐square deviation (RMSD) between the predicted and experimental ligand positions is below 2.0 Å (Bendaas et al. 2024).

#### 2.8.4 Molecular Docking

The phytoconstituents and standard drugs were docked into the active sites of the enzymes defined based on the co‐crystallized ligands using the MOE Dock tool. The Triangular Matcher was maintained as a placement method, with London dG and WSA/GBVI dG as scoring and rescoring functions. For each compound, 10 poses were generated, and five were retained for analysis. The best‐docked poses were selected based on the highest binding affinity and the most favorable interactions.

### 2.9 Statistical Analysis

The results are expressed as the mean ± standard deviation (SD) from three independent parallel analyses. Statistical significance among the means was determined using ANOVA, followed by Tukey's Honest Significant Difference (HSD) test, performed with STATISTICA 10.0 software (StatSoft Inc., USA). A significance threshold of 5% (*p* < 0.05) was applied. To assess the similarity of leaf extracts from various varieties based on their phenolic compounds and biological activities, principal component analysis (PCA) was performed. Additionally, correlations between these variables were analyzed using Pearson's correlation method at a 95% confidence level.

## 3 Results

### 3.1 Phytochemical Analysis

Phytochemical analysis is a crucial preliminary step for identifying constituents known for their biological activities and medicinal benefits. The determination of TPC and TFC contained in VLEs was carried out using colorimetric methods. The findings are illustrated in Table 2.

**TABLE 2:** Content of phenolic compounds and flavonoids in vine leaf extracts.

Samples	TPC ± SD (mg GAE/g dw)	TFC ± SD (mg QE/g dw)
Chikki	87.45 ± 0.19^c^	54.82 ± 0.84^d^
Azorith‐fereinguel	102.49 ± 2.93^d^	75.90 ± 2.55^e^
Bezoul El‐khadem d'Algérie	51.64 ± 2.05^b^	31.91 ± 1.70^b^
Amesski El Hamma	100.14 ± 2.22^d^	42.1 ± 2.08^c^
Hasram	13.62 ± 1.19^a^	21.52 ± 1.80^a^

*Note:* Within a column, mean values labeled with identical superscript letters (^a–c^) are not significantly different, while those with distinct letters differ significantly (*p* < 0.05), based on Tukey's HSD post hoc analysis.

Abbreviations: dw, dry weight; GAE, gallic acid equivalent; QE, quercetin equivalent.

The methanolic extracts of Algerian grapevine leaves show significant variability in TPC and TFC across different cultivars. Among the tested varieties, the Azorith‐fereinguel cultivar exhibited the highest concentrations, with TPC and TFC values of 102.49 ± 2.93 mg GAE/g and 75.90 ± 2.55 mg QE/g, respectively. It was followed by Amesski El Hamma cultivar (100.14 ± 2.22 mg GAE/g TPC and 42.1 ± 2.08 mg QE/g dw TFC) and Chikki cultivar (87.45 ± 0.19 mg GAE/g dw TPC and 54.82 ± 0.84 mg QE/g dw TFC). In contrast, Bezoul El‐khadem d'Algérie displayed lower levels (51.64 ± 2.05 mg GAE/g dw TPC and 31.91 ± 1.70 mg QE/g dw TFC), while Hasram had the lowest content (13.62 ± 1.19 mg GAE/g dw TPC and 21.52 ± 1.80 mg QE/g dw TFC).

Qualitative analysis of methanolic grape leaf extracts was performed using LC/MS. Phenolic compounds were identified by comparing their mass spectra and retention times with those of 35 authentic standards available in the laboratory. Quantification was carried out using equations derived from standard calibration curves, following dilution of the plant extract when necessary. Table 3 presents the findings of the LC/MS analysis.

**TABLE 3:** Phenolic compounds derived from methanolic extracts of indigenous Algerian *V. vinifera* .

			Phenolic compounds quantity (mg/kg)				
Peak	Chemical Compounds	RT (min)	Chikki	Azorith‐fereinguel	Hasram	Amesski El Hamma	Bezoul El‐khadem d'Algérie
1	Shikimic acid	2.208	1843.42	1936.46	ND	1174.91	ND
2	Gallic acid	3.411	18.98	43.57	ND	ND	65.49
3	Caffeine	10.035	ND	432.52	213.65	5.76	ND
4	Caffeic acid	10.220	51.13	65.35	11.75	62.07	31.09
5	Hydroxybenzaldehyde	10.307	2.09	8.77	ND	3.99	4.07
6	Rutin	11.818	2022.04	2672.35	442.64	1218.90	1285.00
7	Polydatin	11.182	109.82	186.54	103.32	80.09	177.92
8	Resveratrol	11.183	396.68	245.64	185.50	74.30	157.89
9	trans‐ferulic acid	12.342	55.99	147.65	35.78	111.12	30.96
10	o‐coumaric acid	11.565	0.94	0.871	ND	2.66	0.15
11	Isoquercitrin	11.865	4174.97	14324.74	3388.99	12030.14	1637.93
12	Hesperidin	11.811	4733.45	3657.34	1037.45	2886.32	2967.69
13	Quercetin‐3‐D‐xyloside	12.175	ND	528.74	56.84	332.25	606.86
14	Quercetin	13.072	144.10	487.63	173.52	323.02	325.23
15	Kaempferol	13.623	9.00	34.72	4.22	16.52	13.15
16	Chrysin	14.753	5.44	8.342	ND	ND	7.66
17	Diosgenin	20.483	0.36	0.342	ND	0.36	0.40

Seventeen compounds were identified in grapevine leaf extracts, with flavonols emerging as the most abundant class. Among the flavonols, isoquercitrin was particularly prominent, especially in the Azorith‐fereinguel variety (14324.7 mg/kg) and the Amesski El Hamma variety (12030.1 mg/kg). Other cultivars such as Chikki, Hasram, and Bezoul El‐khadem d'Algérie also showed notable concentrations of this compound.

Hesperidin was also identified as a major flavonol across all grapevine varieties examined. The highest concentrations were found in the white Azorith‐fereinguel (4733.4 mg/kg) and red Chikki (3657.3 mg/kg) varieties. Notably, hesperidin levels were not dependent on grape color, as substantial amounts were detected in both white and red cultivars.

Quercetin was detected in all varieties, with concentrations ranging from 144.1 mg/kg in the Chikki variety to 487.6 mg/kg in the Azorith‐fereinguel variety. Similarly, rutin was present in all grape varieties, with concentrations ranging from 442.6 mg/kg in the Hasram variety to 2672.3 mg/kg in the Azorith‐fereinguel variety. The concentration of rutin did not appear to be influenced by the grape variety's color, as similar levels were found in both the white Azorith‐fereinguel and the red Chikki varieties.

Among the phenolic acids, shikimic acid was the most prevalent, showing particularly high concentrations in the Azorith‐fereinguel variety (1936.4 mg/kg), followed by Chikki (1843.4 mg/kg) and Amesski El Hamma (1174.9 mg/kg). In contrast, this compound was absent in both the Bezoul El‐khadem d'Algérie and Hasram varieties. Resveratrol showed moderate concentrations, with levels of 396.6 mg/kg in the Chikki variety and 245.6 mg/kg in the Azorith‐fereinguel variety.

**FIGURE 1:**
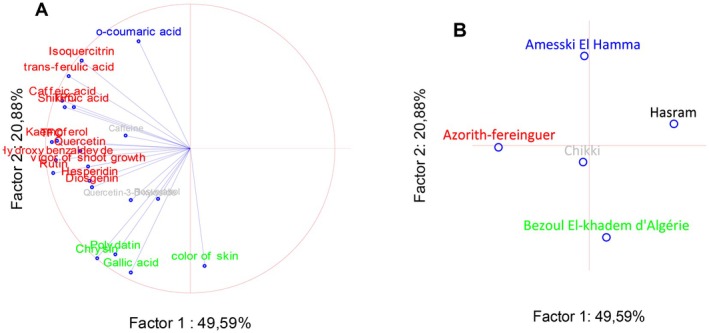
Chemical diversity of Algerian autochthonous *V. vinifera* using the PCA approach.

The PCA analysis of phenolic compound data led to the recognition of five distinct VLE types (Figure 1). On the negative axis of PC1, the first group includes the Azorith‐fereinguel variety, distinguished by its exceptional richness in secondary metabolites. The predominant metabolites in this variety include caffeic acid, shikimic acid, hydroxybenzaldehyde, rutin, quercetin, isoquercitrin, and trans‐ferulic acid. Conversely, the positive side of PC1 encompasses the second group, represented by the Hasram variety, which synthesizes these compounds in substantially lower quantities. The third group, located on the positive axis of PC2, consists of cultivars with deeply pigmented berries (red Bezoul El‐Khadem d'Algérie and black Chikki), characterized by elevated concentrations of gallic acid, chrysin, and polydatin. In contrast, the fourth group, positioned on the negative side of PC2, includes the Amesski El Hamma variety, distinguished by its high levels of coumaric acid.

### 3.2 Biological Activities of the Grapevine Leaves Extracts

#### 3.2.1 Antioxidant Capacity

The antioxidant potential of leaf extracts from five grapevine varieties was evaluated using three in vitro assays (DPPH, ABTS, and reducing power, RP). The findings are presented in Table 4.

**TABLE 4:** Antioxidant activities of grape leaf extracts and drug standards.

Samples/Standards	DPPH scavenging, IC_50_ (μg/mL)	ABTS^+^ scavenging, IC_50_ (μg/mL)	Reducing power, A_0.5_ (μg/mL)
Chikki	6.24 ± 0.29^d^	126.61 ± 6.75^e^	104.96 ± 4.59^c^
Azorith‐fereinguel	3.93 ± 0.13^b^	43.61 ± 4.96^b^	75.12 ± 6.64^b^
Bezoul El‐khadem d'Algérie	7.62 ± 0.31^d^	82.63 ± 9.91^c^	101.47 ± 2.68^c^
Amesski El Hamma	7.15 ± 0.23^d^	75.52 ± 9.78^c^	102.94 ± 0.37^c^
Hasram	4.80 ± 0.16^c^	104.50 ± 3.68^d^	117.65 ± 4.20^d^
BHA	2.72 ± 0.21^b^	11.97 ± 0.08^a^	56.90 ± 1.20^a^
BHT	17.54 ± 1.51^e^	20.45 ± 1.14^a^	63.23 ± 2.36^a^
α‐Tocopherol	1.99 ± 0.10^a^	50.06 ± 1.11^b^	106.40 ± 3.24^c^

*Note:* Within a column, mean values labeled with identical superscript letters (^a–e^) are not significantly different, while those with distinct letters differ significantly (*p* < 0.05), based on Tukey's HSD post hoc analysis.

Abbreviations: dw, dry weight; GAE, gallic acid equivalent; QE, quercetin equivalent.

The DPPH assay revealed IC_50_ values ranging from 3.93 μg/mL to 7.62 μg/mL for the plant extracts. By comparison, the standard controls showed IC_50_ values ranging from 1.99 μg/mL (α‐tocopherol) to 17.54 μg/mL (BHT). The Azorith‐fereinguel variety exhibited the highest capacity to neutralize DPPH radicals, with an IC_50_ of 3.93 μg/mL. This value was similar (*p* < 0.001) to that of the standard BHA (2.72 μg/mL), significantly lower than BHT (17.54 μg/mL), and nearly half the efficacy of α‐tocopherol (1.99 μg/mL). The Hasram variety demonstrated an IC_50_ of 4.80 μg/mL.

Parallel evaluation with the ABTS assay revealed IC_50_ values ranging from 43.61 to 126.61 μg/mL. The Azorith‐fereinguel variety again manifested the highest efficacy (IC_50_ = 43.61 μg/mL), closely approximating that of the standard (50.06 μg/mL). Amesski El Hamma and Bezoul El‐khadem d'Algérie also demonstrated notable antioxidant strengths, while Hasram and Chikki showed attenuated activities.

The reducing power of grape leaves was evaluated as an indicator of electron–donation capacity. The Azorith‐fereinguel extract displayed the highest activity (*A*_0.5_ = 75.12 μg/mL), surpassing BHT (63.23 μg/mL) but remaining lower than α‐tocopherol (106.4 μg/mL). The other varieties showed moderate reducing power compared to α‐tocopherol, though their activity was weaker than that of BHA and BHT.

#### 3.2.2 Antidiabetic Activity

The enzymatic inhibitory activities of grape leaf extracts were assessed using in vitro α‐amylase and α‐glucosidase assays, with results summarized in Table 5.

**TABLE 5:** Enzyme inhibitions of grape leaves extracts and acarbose.

Samples/Standards	*α*‐amylase inhibition, IC_50_ (μg/mL)	α‐glucosidase inhibition, IC_50_ (μg/mL)
Chikki	35.39 ± 0.72^b^	36.27 ± 2.47^d^
Azorith‐fereinguel	22.46 ± 2.46^a^	25.30 ± 3.52^b^
Bezoul El‐khadem d'Algérie	68.38 ± 3.18^c^	18.67 ± 1.12^a^
Amesski El Hamma	32.62 ± 0.45^b^	33.34 ± 0.72^c^
Hasram	36.96 ± 3.26^b^	42.52 ± 1.76^d^
Acarbose	18.40 ± 3.27^a^	27.65 ± 2.41^b^

*Note:* Within a column, mean values labeled with identical superscript letters (^a–d^) are not significantly different, while those with distinct letters differ significantly (*p* < 0.05), based on Tukey's HSD post hoc analysis.

For α‐amylase inhibition, the Azorith‐fereinguel variety exhibited an IC_50_ value of 22.46 μg/mL, which was not significantly different (*p* > 0.05) from that of the reference compound acarbose (18.40 μg/mL). This was followed by the Amesski El Hamma, Chikki, and Hasram varieties, while Bezoul El‐khadem d'Algérie demonstrated comparatively lower inhibitory activity.

For α‐glucosidase inhibition, the Bezoul El‐khadem d'Algérie leaf extract exhibited an IC_50_ value of 18.67 μg/mL, surpassing that of acarbose (27.65 μg/mL). The Azorith‐fereinguel extract also demonstrated substantial α‐glucosidase inhibition (IC_50_ = 25.30 μg/mL). Other varieties showed moderate inhibitory activity, with IC_50_ values slightly higher than that of the standard.

#### 3.2.3 Antibacterial Activity

The antibacterial efficacy of the five crude leaf extracts was assessed against two Gram‐negative ( *E. coli* , *P. aeruginosa* ) and two Gram‐positive ( *S. aureus* , *B. cereus* ) bacterial strains using the broth microdilution assay. Minimum Inhibitory Concentrations (MICs) are detailed in Table 6. Inhibitory activity was observed against all target pathogens, yielding MIC values ranging from 64 to 256 μg/mL. Notably, these MICs exceeded the potency typically demonstrated by reference antibiotic standards, whose MICs ranged from 128 to > 1024 μg/mL.

**TABLE 6:** Minimum inhibition concentrations of grape leaves extracts and antibiotics.

Samples/Antibiotics	Gram negatif bacterial		Gram pozitif bacterial	
	*E. coli* (ATCC25922)	*P. aeruginosa* (ATCC15442)	*S. aureus* (ATCC25213)	*B. cereus* (CCM99)
Chikki	128	128	128	128
Azorith‐fereinguel	64	64	64	64
Bezoul El‐khadem d'Algérie	64	64	64	64
Amesski El Hamma	64	64	64	64
Hasram	256	128	128	256
Ampicillin	256	256	512	256
Chloramphenicol	> 1024	> 1024	> 1024	> 1024
Ofloxacin	128	128	128	128

Abbreviation: MIC, minimum inhibitory concentration (μg/mL).

### 3.3 Chemometric Analysis

**FIGURE 2:**
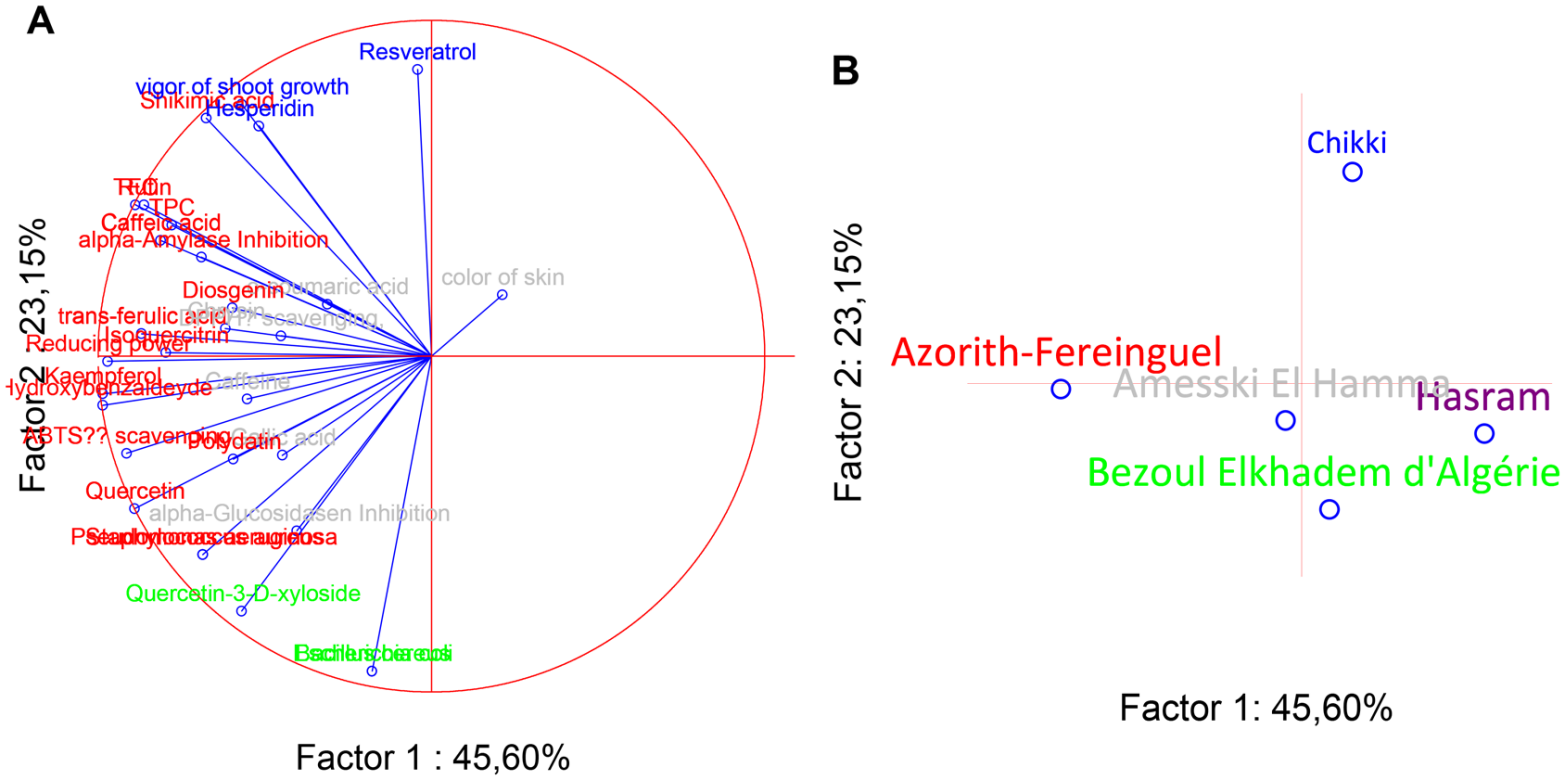
The results of chemometric analysis using the PCA approach. (A) Project plan (1/2) of variables; (B) Project plan (1/2) of varieties.

A full dataset comprising 27 variables characterizing the phytochemical profiles, biological activities, and key morphological traits of the five cultivars was subjected to PCA. The first two principal components explained 68.75% of the total variance. As illustrated in Figure 2, bioactivity‐related parameters (α‐amylase, ABTS, reducing power, and antibacterial activities against *P. aeruginosa* and *S. aureus* ) clustered closely with the chemical profile of the Azorith‐fereinguel cultivar, characterized by high levels of TPC, TFC, quercetin, rutin, polydatin, hydroxybenzaldehyde, kaempferol, isoquercitrin, caffeic acid, trans‐ferulic acid, shikimic acid, and diosgenin. Antibacterial activity against *E. coli* and *B. cereus* was associated with quercetin‐3‐D‐xyloside, detected in high quantities in the Bezoul El‐khadem d'Algérie cultivar. DPPH scavenging and α‐glucosidase inhibitory activities were linked to the chemical profile of the Amesski El Hamma cultivar, which exhibited notable concentrations of caffeine, caffeic acid, o‐coumaric acid, and chrysin.

Pearson's correlation analysis revealed a strong positive correlation between α‐glucosidase inhibitory activity and gallic acid content (*r* = 0.9415, *p* = 0.017). Caffeine exhibited a significant correlation with DPPH radical scavenging activity (*r* = 0.9747, *p* = 0.005). Hydroxybenzaldehyde showed significant positive correlations with both reducing power (*r* = 0.9635, *p* = 0.008) and ABTS radical scavenging capacity (*r* = 0.9325, *p* = 0.021). The ABTS assay exhibited a strong intercorrelation with reducing power (*r* = 0.9245, *p* = 0.016). Robust positive correlations were also found with kaempferol (*r* = 0.9690, *p* = 0.007) and quercetin (*r* = 0.9531, *p* = 0.012). Quercetin‐3‐D‐xyloside demonstrated significant efficacy against *P. aeruginosa* and *S. aureus* (*r* = 0.8914, *p* = 0.042 for both).

### 3.4 *In‐Silico* Molecular Docking Study

**FIGURE 3:**
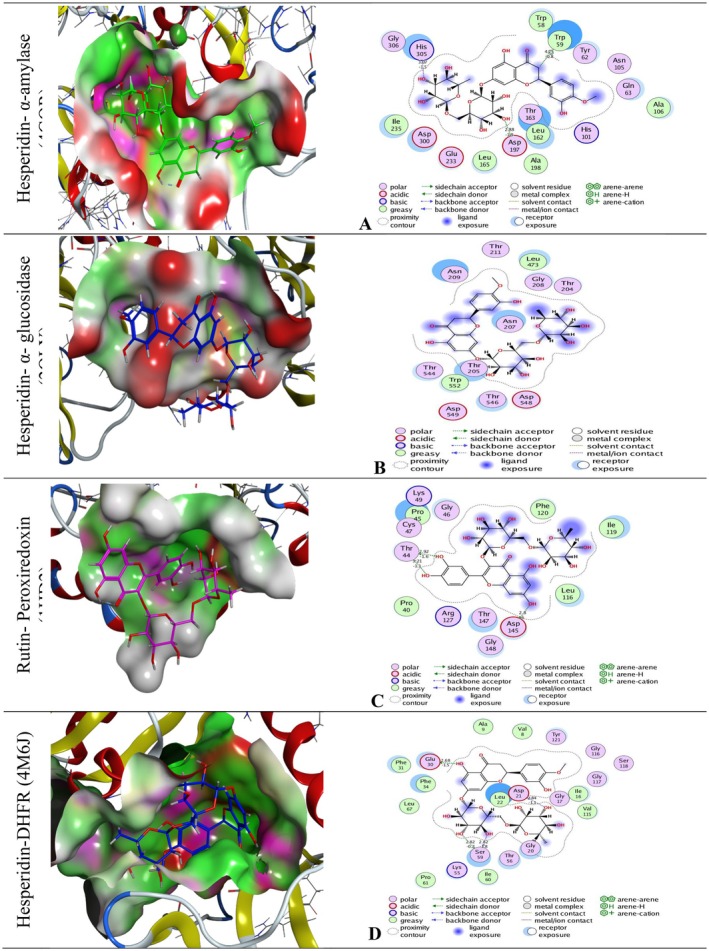
2D and 3D molecular docking simulation studies of the interactions between Hesperidin with the active sites of the receptor of α‐amylase (PDB ID: 4GQR), α‐glucosidase (PDB ID: 2QLY), and Dihydrofolate Reductase (PDB ID: 4M6J), and Rutin with the active site of the receptor of Peroxiredoxin 5 (PDB ID: 1HD2).

Molecular docking analysis was performed on the 17 identified compounds against antidiabetic (4GQR, 2QLY), antioxidant (1HD2), and antibiotic (4M6J) target proteins. The findings are illustrated in Table 7 and Figure 3.

**TABLE 7:** Binding energy (kcal/mol) of *V. vinifera* leaf compounds and synthetic drugs.

PubChem ID	Chemical compounds	Antidiabetic proteins (PDB IDs)		Antioxidant protein (PDB ID)	Antibiotic protein (PDB ID)
		4GQR	2QLY	1HD2	4M6J
5,280,804	Isoquercitrin	−6.4243	−5.1041	−6.1544	−7.4864
10,621	Hesperidin	−7.1694	−5.7186	−5.9601	−8.8342
5,280,343	Quercetin	−5.4464	−4.0225	−4.8400	−5.7298
5,280,805	Rutin	−6.6731	−5.1183	−6.2402	−8.8218
8742	Shimik acid	−4.4032	−3.7769	−4.5740	−4.7345
5,320,863	Quercetin‐3‐D‐xyloside	−6.3705	−4.4329	−5.5313	−7.1674
2519	Caffeine	−4.0231	−4.1978	−4.6840	−5.0295
445,154	Resveratrol	−4.9175	−4.0414	−47,578	−5.5805
5,281,718	Polydatin	−6.5650	−5.2052	−5.5347	−6.8712
445,858	trans‐ferulic acid	−4.4995	−3.8221	−4.5045	−5.0130
689,043	Caffeic acid	−4.6468	−3.8255	−4.6801	−4.9956
370	Gallic acid	−4.3562	−3.7083	−4.4975	−4.69
5,280,863	Kaempferol	−5.2124	−4.7247	−4.8826	−6.1109
11,020,635	Hydroxybenzaldehyde	−4.8435	−3.9656	−4.8895	−5.4695
5,281,607	Chrysin	−4.9658	−3.8866	−4.8610	−5.7688
99,474	Diosgenin	−5.7369	−4.6546	−4.9774	−7.0940
41,774	Acarbose	−8.0157	−6.2333	/	/
8564	BHA	/	/	−4.4413	/
31,404	BHT	/	/	−4.5847	/
14,985	α‐Tocopherol	/	/	−5.8758	/
4583	Ofloxacin	/	/	/	−6.7892
5959	Chloramphenicol	/	/	/	−6.3546
6249	Ampicilline	/	/	/	−6.1697

The binding energy scores for α‐amylase ranged from −4.0231 to −7.1694 kcal/mol, and for α‐glucosidase from −3.7083 to −5.7186 kcal/mol; for Peroxiredoxin5, scores ranged from −4.6801 to −6.2402 kcal/mol. Among the identified compounds, hesperidin, rutin, isoquercitrin, and polydatin exhibited the most favorable binding scores. Hesperidin showed binding affinities of −7.1694 kcal/mol for α‐amylase and −5.7186 kcal/mol for α‐glucosidase. In the α‐amylase binding site (Figure 3A), hesperidin formed two hydrogen bonds (His305, Asp197) and one H‐π interaction (Trp59), along with hydrophobic and electrostatic interactions. In the α‐glucosidase binding site (Figure 3B), hesperidin mediated only hydrophobic and electrostatic interactions.

All major compounds exhibited stronger binding affinity with Peroxiredoxin 5 than the antioxidant drugs BHA, BHT, and α‐tocopherol. Rutin showed a binding energy of −6.24 kcal/mol, forming hydrogen bonds with Thr44 (two bonds) and Asp145, and hydrophobic interactions with Pro40, Pro45, Phe120, Leu116, and Ile119 (Figure 3C).

Against dihydrofolate reductase (DHFR, PDB ID: 4M6J), hesperidin demonstrated the most favorable docking score of −8.8342 kcal/mol, forming four hydrogen bonds with Ser59, Asp21, and Gly30, as well as hydrophobic interactions with Val8, Ala9, Val115, Phe31, Phe34, Leu22, Leu67, Ile16, Ile60, and Pro61 (Figure 3D).

## 4 Discussion

This study represents the first phytochemical screening of methanolic extracts from indigenous Algerian grape leaves. The high phenolic concentrations observed likely result from methanol's polarity, which efficiently extracts phenolic compounds, a finding consistent with previous research on Algerian grapevine varieties (Ferhi et al. 2019). Moreover, methanol–water mixtures have been proven to enhance extraction efficiency and selectivity (Bendrihem et al. 2024). Compared to other Algerian varieties such as Ahmar‐Bou‐Amar (63.9 mg GAE/g TPC, 42.2 mg QE/g TFC) and Tizourine Bou Afraraet (53.6 ± 2.1 mg GAE/g TPC, 32.4 ± 0.8 mg QE/g TFC) (Djemaa‐Landri et al. 2020), the Azorith‐fereinguel, Amesski El Hamma, and Chikki cultivars exhibited notably higher polyphenolic richness. Additionally, the TPC values of Algerian cultivars surpassed those reported for Serbian vine leaves (27–76 mg GAE/g) (Pantelić et al. 2017). However, certain Italian cultivars, including Arvino (200.6 mg/g), Greco Nero (294.5 mg/g), and Nocera (201.6 mg/g), displayed substantially higher concentrations (Loizzo et al. 2019). Generally, variations in phenolic levels can be attributed to genetic differences (Fernandes et al. 2013), extraction methodologies (Djemaa‐Landri et al. 2020), and sample collection periods (Katalinić et al. 2013).

The identification of 17 phenolic compounds, with flavonols as the most abundant class, is consistent with previous reports (Katalinić et al. 2013; Pantelić et al. 2017; Ahmedy et al. 2025). Rana et al. (2022) reported that isoquercitrin stands out as one of the major compounds found in grapevine leaves, highlighting significant variation in phenolic composition across different grape varieties. Baroi et al. (2022) also reported hesperidin as a major compound in the Muscat variety. The prominence of hesperidin suggests it likely contributes significantly to the bioactive properties of these grape varieties. Quercetin levels were consistent with previous studies (Djemaa‐Landri et al. 2020), and rutin concentrations aligned with Jordão et al. (2019), who reported high rutin in both red and white varieties. Shikimic acid, a key precursor in flavonoid biosynthesis (Acero et al. 2025), was abundant in several varieties, indicating substantial contribution to chemical diversity.

Regarding the PCA findings, the high‐altitude Azorith‐fereinguel variety exhibits enhanced biosynthetic capacity for flavonoids, which serve critical photoprotective functions against ultraviolet radiation. Flavonoids play pivotal roles in modulating electron transport during photosynthesis and protecting leaf tissue from UV‐induced oxidative stress (Ghitti et al. 2022). Furthermore, flavonoid biosynthesis constitutes a broad‐spectrum stress response mechanism induced by both biotic and abiotic stimuli (Pucker et al. 2020). These compounds also function as phytoalexins, offering defense against microbial attacks (Corradini et al. 2011; Tiku 2020). Comprehensively characterizing the phenolic composition of *V. vinifera* leaves is vital not only for potential applications in human nutrition but also for differentiating grapevine genotypes and geographic origins. Given their stability and varietal specificity, phenolic compounds constitute reliable chemotaxonomic markers for grapevine classification and traceability.

The observed antioxidant properties may be linked to the high polyphenol content of grape leaves, including flavonoids and other phenolic compounds (Farhadi et al. 2016; Samoticha et al. 2017; Loizzo et al. 2019), aligning with previous reports by Khan et al. (2021) and Deliorman Orhan et al. (2009). Notably, the Hasram variety demonstrated significant antioxidant activity (IC_50_ = 4.80 μg/mL) despite its relatively modest total phenolic and flavonoid contents. This apparent discrepancy suggests that antioxidant efficacy depends not solely on the quantity of polyphenols but also on their qualitative composition and synergistic interactions. In particular, the exceptional performance of Hasram extracts may arise from synergy among minor phenolic constituents or the presence of potent non‐phenolic antioxidants—such as ascorbic acid or specific terpenoids—that are not detected by the Folin Ciocalteu method (Rice‐Evans et al. 1997; Skroza et al. 2022). The phenomenon of synergy, wherein the combined activity of multiple compounds exceeds the sum of their individual effects, is well documented (Rice‐Evans et al. 1997; Prior et al. 2005; Pérez‐Jiménez and Saura‐Calixto 2006; Skroza et al. 2022; Breda et al. 2025).

The distinct performance of the extracts in the DPPH and ABTS assays reflects the differing chemical properties of the two radicals. DPPH primarily interacts with hydrophobic antioxidants, limiting its responsiveness to hydrophilic compounds, whereas ABTS detects both hydrophilic and lipophilic antioxidants, thereby offering a broader assessment of antioxidant capacity (Floegel et al. 2011; Danciu et al. 2016; Cano et al. 2023). Although IC_50_ values were generally lower for DPPH than for ABTS, similar trends have been reported by Loizzo et al. (2019) and Murshed et al. (2024). Consistent with comparative phytochemical research, white grape varieties generally possess greater antioxidant activity than red varieties (Fernandes et al. 2013); in this study, Azorith‐fereinguel (white) was followed by Amesski El Hamma (yellow), Hasram (green), and then Chikki and Bezoul El‐khadem d'Algérie (red). These disparities are attributable to inherent varietal characteristics, as all samples were collected under controlled conditions, reinforcing the critical role of genotype in modulating antioxidant profiles.

Regarding antidiabetic activity, the findings substantiate the dual inhibitory capacity of grape leaf extracts against key carbohydrate‐hydrolyzing enzymes, a mechanism recognized as central to the attenuation of postprandial hyperglycemia (Dar et al. 2022; Singh et al. 2023). The observed antidiabetic efficacy is largely attributable to their high flavonoid content, as flavonoids are extensively documented for their ability to inhibit both α‐amylase and α‐glucosidase (Zhu et al. 2020). Notably, Li et al. (2009) identified quercetin, isoquercitrin, and rutin as potent α‐amylase inhibitors, underscoring their therapeutic relevance in mitigating hyperglycemia and obesity‐associated metabolic dysfunctions. Moreover, synergistic interactions among flavonoid constituents may potentiate inhibitory efficacy through complementary mechanisms (Kim 2000). Given the abundance of flavonoids in grape leaf extracts, these bioactive compounds likely constitute the principal contributors to the observed enzymatic inhibition, thereby reinforcing the therapeutic potential of *V. vinifera* leaves as a natural source for managing diabetes mellitus.

Turning to antibacterial activity, previous investigations employing the agar well diffusion method have demonstrated that crude extracts of *V. vinifera* leaves possess broad‐spectrum antimicrobial activity against diverse Gram‐positive and Gram‐negative bacteria (Oskay and Sarı 2007; Ahmad et al. 2014; Selka et al. 2016; Ali et al. 2019; Khan et al. 2021). Although the effectiveness varied among the five varieties, the differences were not statistically significant. As a result, choosing a particular variety as a preferred source of antibacterial chemicals was not feasible. Furthermore, there was no great difference in activity against Gram‐positive and Gram‐negative bacteria, which is typically reported for plant phenolic extracts. These findings are consistent with the phytochemical and bioactivity profiles documented by Katalinić et al. (2013) for ethanolic leaf extracts of six *V. vinifera* varieties, suggesting a multi‐target mechanism. The multi‐target antibacterial action of phenolic compounds is well‐documented and arises from their ability to interact with multiple bacterial cellular components simultaneously (Zhao et al. 2025). Unlike conventional antibiotics that target a single bacterial pathway, phenolic compounds act via multiple concurrent mechanisms: disrupting cell membranes, inhibiting key enzymes (e.g., DHFR), inducing oxidative stress, and suppressing virulence factors such as biofilm formation and quorum sensing (Wróbel et al. 2020; Agu et al. 2023). The major flavonols in our study (hesperidin, rutin, isoquercitrin, and quercetin‐3‐D‐xyloside) exhibit these multi‐target effects: hesperidin disrupts membranes and biofilms (Vijayakumar et al. 2022), rutin reverses antibiotic resistance by inhibiting efflux pumps (Miklasińska‐Majdanik et al. 2023), and quercetin derivatives block DNA replication via gyrase binding (Plaper et al. 2003). Their collective action explains the broad‐spectrum activity across bacterial types and the lack of significant differences among grape varieties, which share qualitatively similar phenolic profiles.

From a food science perspective, the demonstrated antioxidant and antibacterial activities of these extracts suggest practical applications as natural food preservatives to inhibit lipid oxidation and microbial spoilage in food matrices. The potent inhibition of carbohydrate‐hydrolyzing enzymes further positions these phenolic‐rich extracts as promising functional food ingredients for managing postprandial glycemic responses, which is highly relevant to the development of low‐glycemic functional foods. Furthermore, the abundance, renewability, and traditional edible status of grapevine leaves as an agro‐industrial by‐product enhance their appeal for sustainable food systems, offering a low‐cost, naturally sourced alternative to synthetic food additives (e.g., BHA, BHT) while contributing to the valorization of viticultural waste streams (Singh et al. 2023).

Finally, the *in silico* molecular docking study provided mechanistic support for the experimental bioactivities. The more negative the docking score, the stronger the binding affinity, indicating an increased probability of effective interaction (Agu et al. 2023). Hesperidin's binding affinities for α‐amylase and α‐glucosidase, though slightly less potent than acarbose, indicate robust and significant interactions. The molecular interactions observed—hydrogen bonds, hydrophobic, and electrostatic interactions—contribute to the stability of the enzyme–inhibitor complexes, influencing enzymatic activities. These findings are consistent with recent in silico studies demonstrating the strong binding affinity of *V. vinifera* ‐derived compounds, including resveratrol, with various therapeutic targets (Kalyanaraman et al. 2024). All major compounds exhibited stronger binding affinity with peroxiredoxin 5 than standard antioxidant drugs, signifying the important antioxidant activity of *V. vinifera* leaf extracts. Regarding antibacterial activity, inhibition of dihydrofolate reductase (DHFR) is a well‐established strategy in the development of antibacterial agents, as it disrupts cell replication and growth (Wróbel et al. 2020; Bayazeed et al. 2022; Agu et al. 2023; Al‐Mijalli et al. 2025). The higher binding affinities of hesperidin, rutin, isoquercitrin, quercetin‐3‐D‐xyloside, diosgenin, and polydatin to DHFR compared to standard antibiotics provide a theoretical basis for the observed antibacterial activity. Together, these integrated experimental and computational findings establish Algerian *V. vinifera* leaves as a promising source of bioactive phenolic compounds with antioxidant, antidiabetic, and antibacterial properties.

While the in silico docking results provide strong mechanistic support for the observed enzymatic inhibition, it is important to acknowledge that the in vivo bioavailability of polyphenol glycosides such as rutin and hesperidin may be limited by factors including poor intestinal absorption, extensive first‐pass metabolism, and rapid systemic clearance (Amaretti et al. 2015; Hoang et al. 2026). Polyphenol glycosides are often glycosylated by rutinose, which hampers absorption in the small intestine, requiring gut microbiota to release the aglycone for colonic absorption (Amaretti et al. 2015). Future studies should therefore include pharmacokinetic and bioavailability assessments to translate these in vitro findings into practical food and nutraceutical applications.

## 5 Conclusion

This study provides the first integrated phytochemical and pharmacological profiling of five autochthonous Algerian *V. vinifera* leaf cultivars, demonstrating significant interspecific variation in phenolic composition and bioactivity. The Azorith‐fereinguel cultivar emerged as the most promising candidate, exhibiting the highest TPC/TFC, superior antioxidant capacity, potent dual α‐amylase/α‐glucosidase inhibition, and the lowest antibacterial MICs. LC–MS profiling identified isoquercitrin, hesperidin, and rutin as predominant bioactive markers, whose concentrations strongly correlated with enzymatic and microbial inhibition outcomes. In silico docking mechanistically validated these findings, revealing that hesperidin and rutin form stable, multi‐modal interactions with catalytic residues of α‐amylase, α‐glucosidase, Prx5, and bacterial DHFR, thereby rationalizing their antidiabetic, antioxidant, and antibacterial potentials. Crucially, the study establishes a direct structure–activity linkage between specific leaf‐derived phenolics and broad‐spectrum antibacterial efficacy, highlighting their capacity to disrupt microbial membranes and inhibit essential folate metabolism. Collectively, these results position Algerian autochthonous grapevine leaves as a sustainable, high‐value biomass for developing standardized nutraceuticals, functional food ingredients, and natural food preservatives. Future work should focus on in vivo validation, bioavailability enhancement, formulation into food‐compatible matrices, and targeted isolation of lead compounds for both nutraceutical and functional food applications, ultimately translating this sustainable biomass into tangible benefits for human health and food systems.

## Author Contributions

**Khadra Afaf Bendrihem:** methodology, validation, formal analysis, project administration. **Ayomide Victor Atoki:** resources, writing – review and editing. **Mohammed Messaoudi:** data curation, supervision, resources, methodology, funding acquisition, writing – original draft, visualization. **Ahmed Simozrag:** writing – review and editing, software, data curation, supervision. **Ziane Laiadi:** visualization, investigation. **Radhia Belmanaa:** conceptualization, investigation, funding acquisition, formal analysis, software, writing – original draft. **Azzeddine Zeraib:** writing – original draft, funding acquisition, validation, project administration.

## Funding

The authors have nothing to report.

## Conflicts of Interest

The authors declare no conflicts of interest.

## Data Availability

Data involved in the present work are available from the corresponding author upon request.
